# Programmed Cell Death and Aerenchyma Formation in Water-Logged Sunflower Stems and Its Promotion by Ethylene and ROS

**DOI:** 10.3389/fpls.2018.01928

**Published:** 2019-01-09

**Authors:** Xi-Lu Ni, Meng-Yuan Gui, Ling-Ling Tan, Qiang Zhu, Wen-Zhe Liu, Chang-Xiao Li

**Affiliations:** ^1^Breeding Base for State Key Laboratory of Land Degradation and Ecological Restoration of North-western China, Key Lab for Restoration and Reconstruction of Degraded Ecosystem in North-western China of Ministry of Education, Yinchuan, China; ^2^Key Laboratory for the Eco-Environment of the Three Gorges Reservoir Region of the Ministry of Education, College of Life Science, Southwest University, Chongqing, China; ^3^School of Life Science, Northwest University, Xi'an, China; ^4^State Key Laboratory Cultivation Base for Cell Differentiation Regulation, Henan Normal University, Xinxiang, China; ^5^College of Life Science, Qingdao Agricultural University, Qingdao, China

**Keywords:** aerenchyma, *Helianthus annuus*, programmed cell death, Ethylene, reactive oxygen species, waterlogging

## Abstract

Previous studies have shown that waterlogging/ hypoxic conditions induce aerenchyma formation to facilitate gas exchange. Ethylene (ET) and reactive oxygen species (ROS), as regulatory signals, might also be involved in these adaptive responses. However, the interrelationships between these signals have seldom been reported. Herein, we showed that programmed cell death (PCD) was involved in aerenchyma formation in the stem of *Helianthus annuus*. Lysigenous aerenchyma formation in the stem was induced through waterlogging (WA), ethylene and ROS. Pre-treatment with the NADPH oxidase inhibitor diphenyleneiodonium (DPI) partially suppressed aerenchyma formation in the seedlings after treatment with WA, ET and 3-amino-1, 2, 4-triazole (AT, catalase inhibitor). In addition, pre-treatment with the ethylene perception inhibitor 1-methylcyclopropene (1-MCP) partially suppressed aerenchyma formation induced through WA and ET in the seedlings, but barely inhibited aerenchyma formation induced through ROS. These results revealed that ethylene-mediated ROS signaling plays a role in aerenchyma formation, and there is a causal and interdependent relationship during WA, ET and ROS in PCD, which regulates signal networks in the stem of *H. annuus*.

## Introduction

Aerenchyma formation is a major physiological and morphological adaptation of plants to waterlogging or flooding conditions (Jiang et al., 2010). It has long been known that rapidly formed aerenchyma is critical for waterlogged plants in maintaining adequate oxygen supply and overall hypoxia tolerance (Armstrong, 1979; Evans, 2003; Armstrong and Armstrong, 2014). In addition, aerenchyma formation is enhanced with increased soil waterlogging (Das and Jat, 1977; Shiono et al., 2011). For example, in rice (*Oryza sativa*), aerenchyma is well developed in the roots (Joshi and Kumar, 2012), internodes (Steffens et al., 2011) and leaf sheaths (Parlanti et al., 2011). It also has been reported that aerenchyma can develop through programmed cell death (PCD) and lysis in waterlogged non-aquatic plants, such as maize (Drew et al., 1989), pea (Gladish et al., 2006), *Luffa cylindrica* (Shimamura et al., 2007), *Rumex palustris* (Pierik et al., 2009), and wheat (Yamauchi et al., 2014), to name a few. In addition, waterlogging-tolerant soybean genotypes were able to form more than 20% aerenchyma after 7 days of a waterlogging treatment (Thomas et al., 2005; Shimamura et al., 2010). The characteristics of PCD during aerenchyma formation in maize roots was examined, and plasma membrane invagination, small vesicle formation, DNA cleavage, chromatin condensation, organelle retention in the cytoplasm, and cell wall degradation were observed during lysigenous aerenchyma formation under hypoxic conditions (Gunawardena et al., 2001a,b).

It has been showed that hypoxia stimulates ethylene (ET) biosynthesis, and an increase in 1-aminocyclopropane-1 -carboxylic acid (ACC) oxidase and ACC synthase activities have been observed in extracts from hypoxic roots (He et al., 1996; Drew et al., 2000). In water-submerged roots, ethylene rapidly accumulates and plays a role in inducible lysigenous aerenchyma formation in wheat (Yamauchi et al., 2014), maize (He et al., 1996; Gunawardena et al., 2001a), and rice (Steffens et al., 2011; Yamauchi et al., 2015). In addition, in rice stems treated with 150 μM ethephon, the percentage of aerenchyma formation increased from 64.6 to 89.7% after 2 days, and continue increased to nearly 100% after 4 days (Steffens et al., 2011). Increasing direct or indirect evidence suggests that ethylene plays a regulatory role in lysigenous aerenchyma formation (Jackson and Armstrong, 1999; Drew et al., 2000; Evans, 2003). Treatment with inhibitors of ethylene activity or ethylene biosynthesis, such as 1-methylcyclopropene (1-MCP), effectively decrease the amount of aerenchyma formation under hypoxic conditions in rice, maize, arabidopsis and wheat (Jackson et al., 1985; Gunawardena et al., 2001a; Mühlenbock et al., 2007; Rajhi et al., 2011; Steffens et al., 2011; Yamauchi et al., 2015). Therefore, aerenchyma formation in response to submergence or WA is regulated through ethylene.

However, the signal transduction pathways underlying the activation of ethylene signaling and subsequent PCD during aerenchyma formation has not yet been investigated. Indeed, reactive oxygen species (ROS), hydrogen peroxide (H_2_O_2_) and superoxide anion radical (${\text{O}}_{2}^{.-}$) which are produced by either plasma membrane (PM) NAD(P)H oxidase and/or mitochondria, play a role as plant signaling molecules and adjust with an adaptive response to biotic and abiotic stresses (Overmyer et al., 2003; Bailey-Serres and Chang, 2005; Baxter et al., 2014). ROS has the role to regulate ethylene-induced epidermal cell death in rice through an autoamplified signal pathway (Steffens and Sauter, 2009; Steffens et al., 2012). ROS also have been verified as a key signaling molecules during the hypersensitive reaction of lettuce cells in *Pseudomonas syringae* pv *phaseolicola* (Bestwick et al., 1997). In addition, ethylene and ROS have been implicated in the regulation of lysigenous aerenchyma formation of wheat seedlings to adapt oxygen-deficient conditions (Yamauchi et al., 2014).

*Helianthus Annuus*, like most dryland crops, is sensitive to hypoxia condition and appears to significantly decline in yields when the plants were waterlogged (Zhang et al., 2015). It was reported that both waterlogging and ethylene treatments could induce aerenchyma formation in the roots of *H. annuus* (Kawase, 1974, 1981; Kawase and Whitmoyer, 1980). But no published study has yet characterized aerenchyma formation in stem/root of *H. annuus* associated with PCD, and nor the roles of ET and ROS during aerenchyma formation under conditions of waterlogging. We set out to study this phenomenon, in order to understand the mechanisms of aerenchyma formation in sunflower with the aim of improving this crop plant's ability to tolerate waterlogging. We hypothesize that: (1) the involvement of PCD in the process of induced aerenchyma morphogenesis in *H. annuus* by waterlogging condition; and (2) ET and ROS play important roles in inducing lysigenous aerenchyma formation in *H. annuus* stem. In the present study, the characteristics of PCD during inducible aerenchyma formation in the stem of *H. annuus* were investigated using light microscopy, transmission electron microscopy, TUNEL assays, and gel electrophoresis. In addition, we examined the effects on lysigenous aerenchyma formation of ET and its perception inhibitor 1-MCP, the catalase inhibitor 3-amino-1, 2, 4-triazole (AT) and NADPH oxidase inhibitor diphenyleneiodonium (DPI). Taken together, these results showed PCD is involved in aerenchyma formation in waterlogged *H. annuus* stems. Moreover, ethylene-mediated ROS play important roles in triggering PCD occurrence and result in lysigenous aerenchyma formation.

## Materials and Methods

### Plant Material and Growth Conditions

*H. annuus* seeds were sown in a dampened vermiculite medium (with the addition of 150 ml hoagland solution every 3 days) for germination at 26°C. The seedlings were maintained in an illumination incubator (photosynthetically active radiation, 300 μmol/m^2^s) in the condition of 12-h photoperiod and ~70% relative moisture for 15 days. Subsequently, seedlings at 4-leaf stage were transplanted to plastic pots (4 plants per pot, 80 mm width × 100 mm length × 100 mm height). To examine the process of aerenchyma formation, the seedlings were waterlogged to the basal leaf node by submerging the pots in a tank of distilled water for 4 days, so the whole stem was almost under the water level, except for the leaves and stem apex of the seedlings. As a control, the plants were cultured under the same conditions, without flooding.

### Experimental Design

To determine the effects of WA, ET and ROS on the formation of lysigenous aerenchyma, 15-day-old seedlings (4-leaf stage) were transferred to plastic pots, then, these seedlings were divided into four groups: (Group I) This experimental group was designed to examine the role of WA, ET and ROS on lysigenous aerenchyma formation (Table 1). After 1 day of normal growth, the seedlings were treated with WA (the treatment was the same as the seedlings used for aerenchyma induction by waterlogging), 150 μM ET aqueous solution (Xianyang Xiqing Bio. Sci-Tec Co. Ltd., China), 50 mM AT reagent [0.42g AT (Sigma) dissolved in 100 ml dimethyl sulfoxide (DMSO)] and 150 μM ET + 50 mM AT, all for 6 days. The seedlings in the control group were sprayed with distilled water. (Group II) This experimental group was designed to examine the role of WA in lysigenous aerenchyma formation under ET and ROS signals to be suppressed (Table 2). Some of the plants, used as a control, were allowed to grow normally for 1 day by sprayed with distilled water, and then were treated with WA for 6 days; some of the plants were pre-treated with 5 mg/ml 1-MCP aqueous solution (Xianyang Xiqing Bio. Sci-Tec Co. Ltd., China) or 50 μM DPI [3.2mg DPI (Sigma) dissolved in 1 L DMSO] for 1 d, and then were treated with WA for 6 days. (Group III) This group was designed to examine the role of ET in lysigenous aerenchyma formation under ET/ ROS signals to be suppressed (Table 3). Control plants were allowed to grow normally for 1 day by sprayed with distilled water, and then were treated with 150 μM ET for 6 days; some of the plants were pre-treated with 5 mg/ml 1-MCP or 50 μM DPI for 1 d, and then were treated with 150 μM ET for 6 days. Group IV). This experimental group was designed to examine the role of ROS in lysigenous aerenchyma formation under ET/ ROS signals to be suppressed (Table 4). Control plants were allowed to grow normally grow for 1 day, by sprayed with distilled water, and then were treated with 50 mM AT for 6 days. Some of the plants were pre-treated with 5 mg/ml 1-MCP or 50 μM DPI for 1 d, and then were treated with 50 mM AT for 6 days. All of the reagents were prepared fresh and used at room temperature, and the solutions were sprayed only once.

**Table 1:** The experimental design of the role of WA, ET, and ROS in lysigenous aerenchyma formation.

**Number**	**Conditions**	**Growth (for 15d)**	**Pre-treatment (for 1d)**	**Treatment (1-6d)**
1	WA	NG	NG	WA
2	AT	NG	NG	AT
3	ET	NG	NG	ET
4	WA+ET	NG	NG	WA+ET

*AT, 3-amino-1,2,4-triazole; CK, Control; ET, ethephon; NG, normal growth without treatment; WA, waterlogging*.

**Table 2:** The role of WA in lysigenous aerenchyma formation under ET and ROS signals to be suppressed.

**Number**	**Conditions**	**Growth (for 15d)**	**Pre-treatment (for 1d)**	**Treatment (1-6d)**
1	WA	NG	NG	WA
2	WA+1-MCP	NG	1-MCP	WA
3	WA+DPI	NG	DPI	WA

*1-MCP, 1-Methylcyclopropene; CK, Control; DPI, diphenylene iodonium; NG, normal growth without treatment; WA, waterlogging*.

**Table 3:** The role of ET in lysigenous aerenchyma formation under ROS signals to be suppressed.

**Number**	**Conditions**	**Growth (for 15d)**	**Pre-treatment (for 1d)**	**Treatment (1-6d)**
1	ET	NG	NG	ET
2	ET+1-MCP	NG	1-MCP	ET
3	ET+DPI	NG	DPI	ET

*1-MCP, 1-Methylcyclopropene; CK, Control; DPI, diphenylene iodonium; ET, ethephon; NG, normal growth without treatment*.

**Table 4:** The role of ROS in lysigenous aerenchyma formation under ET signals to be suppressed.

**Number**	**Conditions**	**Growth (for 15 d)**	**Pre-treatment (for 1 d)**	**Treatment (1-6 d)**
1	AT	NG	NG	AT
2	AT+1-MCP	NG	1-MCP	AT
3	AT+DPI	NG	DPI	AT

*1-MCP, 1-Methylcyclopropene; AT, 3-amino-1,2,4-triazole; CK, Control; DPI, diphenylene iodonium; NG, normal growth without treatment*.

All treated and control seedlings were sealed in a transparent plastic bag (10 plants from each group) and maintained under the growth conditions described above. During the treatment period, which lasted up to 6 days, changes in aerenchyma size/area in the stems of *H. annuus* seedlings were determined daily using light microscopy (Nikon Eclipse 50i with NikonDS-Fi1 camera, Tokyo, Japan) at a distance of 10 mm above the stem base. The amount of aerenchyma formation in the stem was expressed as the percentage of each cross-section occupied by aerenchyma. Three independent experiments, each with 3 replicates, were performed. Values are expressed as the average of the 9 measurements.

### Light Microscopy and TUNEL and DAPI Assays

The tissue samples, which were waterlogged at different days were cut into small pieces (2–3 mm), and subsequently fixed in 0.1 M phosphate buffer (pH 7.0) containing 2.5% glutaraldehyde at 4°C overnight. After rinsing three times in 0.1 M phosphate buffer (pH 7.0) for 20 min each time, the samples were post-fixed in 0.5% osmium tetroxide (solution in buffer) at 4°C for 3 h. Subsequently, the samples were rinsed three times in 0.1 M phosphate buffer (pH 7.0) for 20 min each time. The samples were dehydrated in series along a gradient of ethanol concentrations (once in 30, 50, 70, 85, and 90% ethanol, and twice in 100% ethanol) for 30 min at each step, with a final change of 1, 2-Epoxypropane, followed by embedding in Epon 812. Semi-thin sections (1–2 μm) were cut using a Reichert-Jung ultramicrotome (Vienna, Austria) and stained with toluidine blue O. The sections were examined and digitally recorded using a Leica microscope (DMLB) equipped with a video camera (Leica, DC 300F, Wetzlar, Germany) (Zhou and Liu, 2011; Ni et al., 2014).

The seedlings waterlogged at different days were used for *in situ* terminal deoxynucleotidyl transferase-mediated dUTP nick end labeling (TUNEL) assays and 4′,6-diamidino-2-phenylindole dihydrochloride (DAPI) staining;.Samples were prepared according to the protocol of Sarkar and Gladish (2012). The samples were fixed in 4% paraformaldehyde overnight, followed by dehydration in a graded ethanol series and embedding in paraffin. The TUNEL and DAPI assays were performed according to the manufacturer's instructions of the *in situ* Apoptosis Detection Kit (TaKaRa, Dalian, China) and were examined using a fluorescence microscope (Leica DMLB microscope equipped with Leica DC 300 F camera). Each sample had three replicates, and 10 microscopic fields (20 × objective lens) were observed for each replicate.

### DNA Extraction and Gel Electrophoresis

Genomic DNA was isolated from the waterlogged seedlings from the first to the fourth day, and untreated seedlings were used as a control. The preparation was done according to the protocol of Ni et al. (2014). Approximately 60 mg of tissues were obtained from seedlings at different developmental stages and immediately frozen in liquid nitrogen, followed by grinding to an ultrafine texture using a mortar and pestle. The DNA isolation was performed using the Universal Genomic DNA Extraction Kit Ver. 3.0 (TaKaRa, Dalian, China) according to the manufacturer's instructions. To observe DNA fragmentation, the samples were run with a 2,000 bp ladder on a 1.0% ethidium bromide agarose gel for approximately 1.5 h at a constant 60 V and subsequently photographed using a gel imaging system. This test was repeated three times.

### Transmission Electron Microscopy

Ultrastructural analysis of tissue samples were carried out as previously reported by Ni et al. (2014). The samples waterlogged at different days were fixed in 0.1 M phosphate buffer (pH 7.0) containing 2.5% glutaraldehyde at 4°C for 4 h. After being rinsed three times in 0.1 M phosphate buffer (pH 7.0) for 30 min each time, the samples were fixed in 1% osmium tetroxide overnight at 4°C, followed by rinsing three times in 0.1 M phosphate buffer (pH 7.0) for 30 min each time. The samples were dehydrated in a series along a gradient of ethanol concentrations (once in 30, 50, 70, 85, and 90% ethanol, and twice in 100% ethanol) at 4°C, followed by a final change in 1, 2- epoxypropane and embedding in Epon 812. The samples were cut into 70–90 nm sections using a Leica EM UC6 ultramicrotome (Vienna, Austria). The sections were mounted on copper grids and stained with uranyl acetate and lead citrate. Observations were made using an H-7650 transmission electron microscope (Hitachi, Japan). Each sample had three replicates.

### Statistical Analysis

Data were collected according to “Observation Methodology for Long-term Forest Ecosystem Research” of the National Standards of China (GB/T 33027-2016). Significant differences between the means ± standard error were calculated using oneway analysis of variance (ANOVA) and *post-hoc* Tukey's test. All data were illustrated as mean ± standard error. Analyses were carried out using SPSS Statistics Version 19 software (IBM Software, New York, NY, USA).

## Results

### Lysigenous Aerenchyma Formation Under Waterlogging Condition

**Figure 1:**
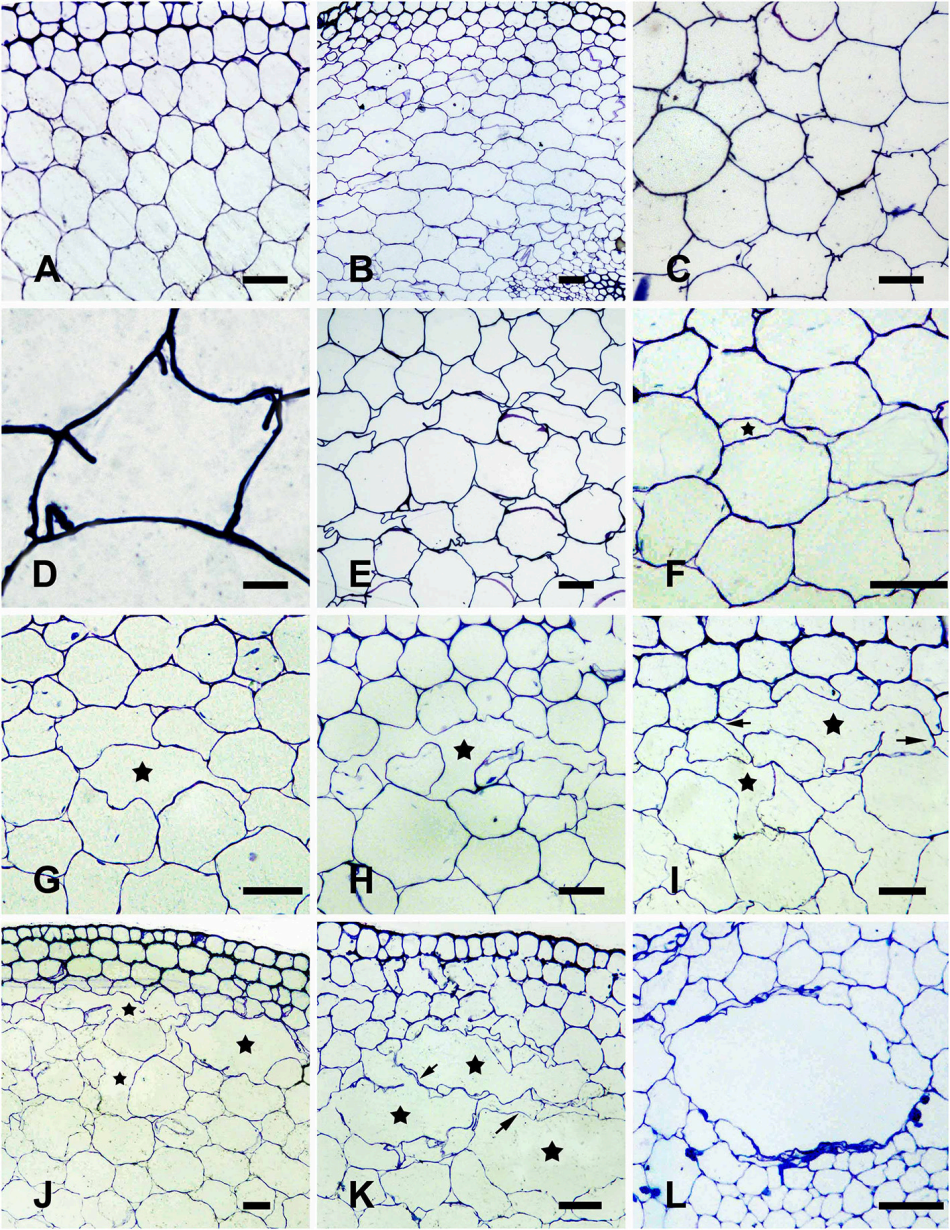
Aerenchyma formation in *H. annuus* stems is induced through waterlogging. **(A)** Normal growth plant without WA treatment (control), showing little intercellular space in the cortex parenchyma; **(B–L)** WA-induced aerenchyma formation in seedlings waterlogged at different period of time. **(B–D)** Early phase of aerenchyma formation in the seedlings after 12 h of WA treatment; **(B)** Parenchymal cells become flattened; **(C)** Cell wall infolding (arrow) is observed; **(D)** Enlargement of **(C)**. **(E–G)** Formation phase of aerenchyma formation; **(E)** The cell wall distortion and plasmolysis were observed after 24 h WA treatment; **(F)** Cell walls become sunken and little intercellular space (star) is observed; **(G)** The volume of intercellular space (star) is increased by evident sunken cell walls after 2 days WA treatment; **(H–K)** Expansion phase of aerenchyma in the seedlings after 3 days of WA treatment; **(H)** The surrounding cells (arrow) of aerenchyma (star) begin to degrade; **(I)** The cells around the cavity continue to degrade (arrow) to enlarge the cavity (star); **(J)** The cells (arrow) between adjacent aerenchyma begin to degrade; **(K)** Neighboring aerenchyma cavities fuse by cells fully degraded (arrow); **(L)** Mature phase of aerenchyma in the seedlings after 4 days WA (star). There are three different segments was analyzed for each state. Bars: *D* = 10 μm, the others = 50 μm.

The parenchymal cells in the cortex of *H. annuus* stems were round under normal growth conditions, without treatment (control; Figure 1A). After 12 h WA, flattened parenchymal cells (Figure 1B) and cell wall infolding (Figures 1C,D) were occasionally observed. An additional 12 h of WA resulted in conspicuously deformed cells, and the cell wall was obviously distorted (Figure 1E), representing the early phase of aerenchyma formation. At 2 days after WA, a small intercellular space was observed between the cells in certain areas of the cortex (Figure 1F), and the side of the cell toward the intercellular space was sunken, thereby increasing the area of the space (Figure 1G). This process represented the formation cavity stage of aerenchyma formation. After 3 days WA, the cells surrounding the intercellular space gradually and distinctly shrunk, and some special cells were degraded, forming a small cavity in the cortex (Figure 1H). The cells surrounding the cavity continued to degrade, tangentially enlarging the cavity (Figure 1I). Certain cells between neighboring aerenchyma cavities began to degrade at this time, and subsequently the neighboring aerenchyma cavities fused together (Figure 1J). After the cells were completely degraded, the neighboring aerenchyma cavities were completely fused, with a large amount of cellular debris remaining in the middle of the merged cavity (Figure 1K), representing the expanding cavity stage of aerenchyma formation. By the fourth day of WA, several layers of cells around the cavity continued to degrade, further increasing the area of the cavity (Figure 1L), representing the mature cavity stage of aerenchyma formation.

**Figure 2:**
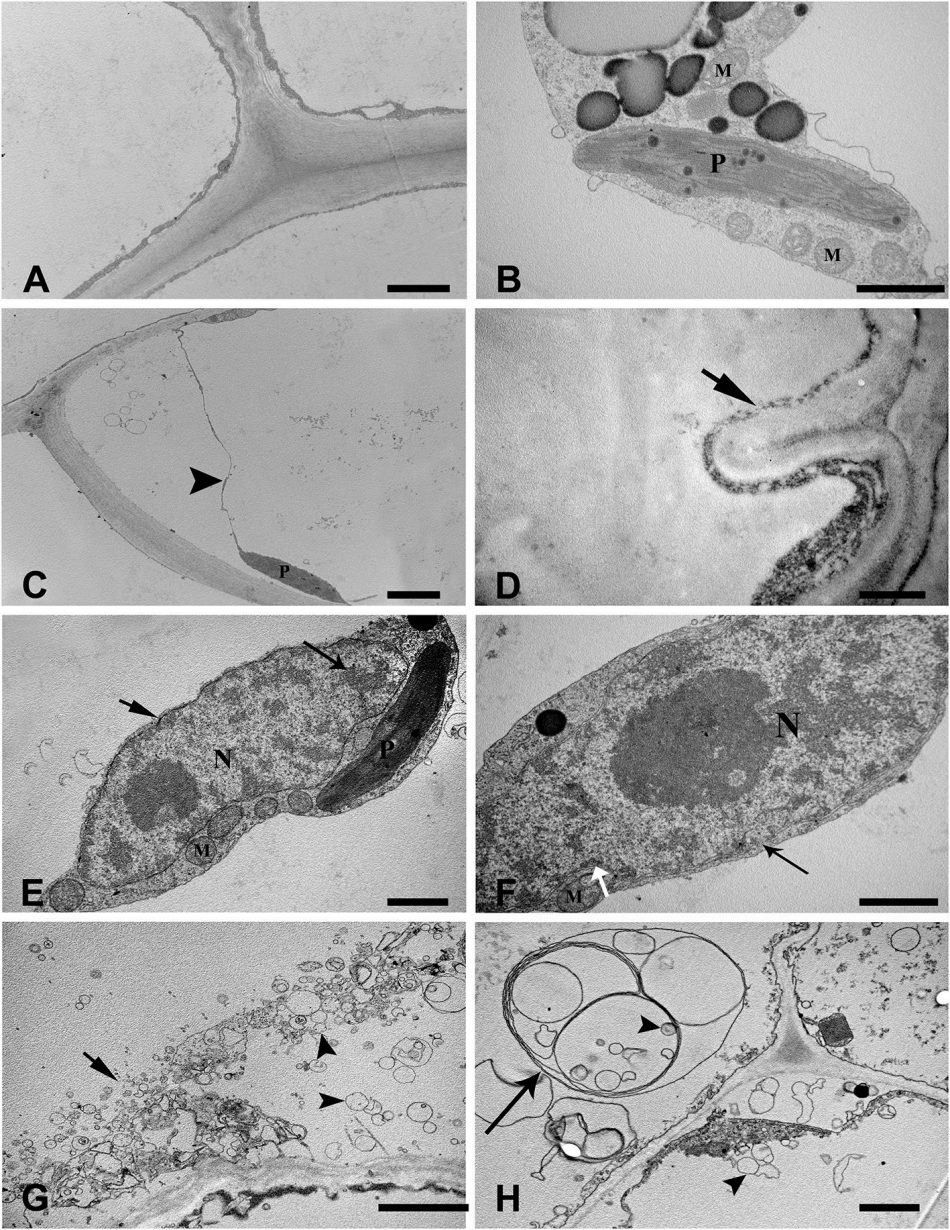
Ultrastructure of aerenchyma during the early phase of formation in *H. annuus* stems. **(A,B)** Ultrastructure of the control, without WA treatment. **(A)** Normal ultrastructure with intact cell walls, cytoplasm and large central vacuoles. **(B)** Intact organelles in the cytoplasm, including mitochondria and plasmids. **(C,D)** Cortical cells at the early pre-cavity stage displayed plasmolysis (arrowhead) **(C)** and cell wall infolding (arrow) **(D)** after 12 h of WA treatment. **(E)** At 1 days after WA, condensed chromatin appeared in the nuclei (long arrow), and the nuclear envelope was heavily stained (arrow). **(F)** Hallmarks of nuclei degradation with nuclear invaginations (white arrow). **(G)** After 2 days of WA, tonoplast rupture (arrow) and lots of vesicles appearance (arrowhead). **(H)** Multilamellar structures (long arrow), in which some enveloped vesicles (arrowhead) were present in the cytoplasm. There are three different pictures was analyzed for each state. Bar: **(A,C,G)** = 2 μm, **(B,E,F,H)** = 1 μm, the others = 0.5 μm.

**Figure 3:**
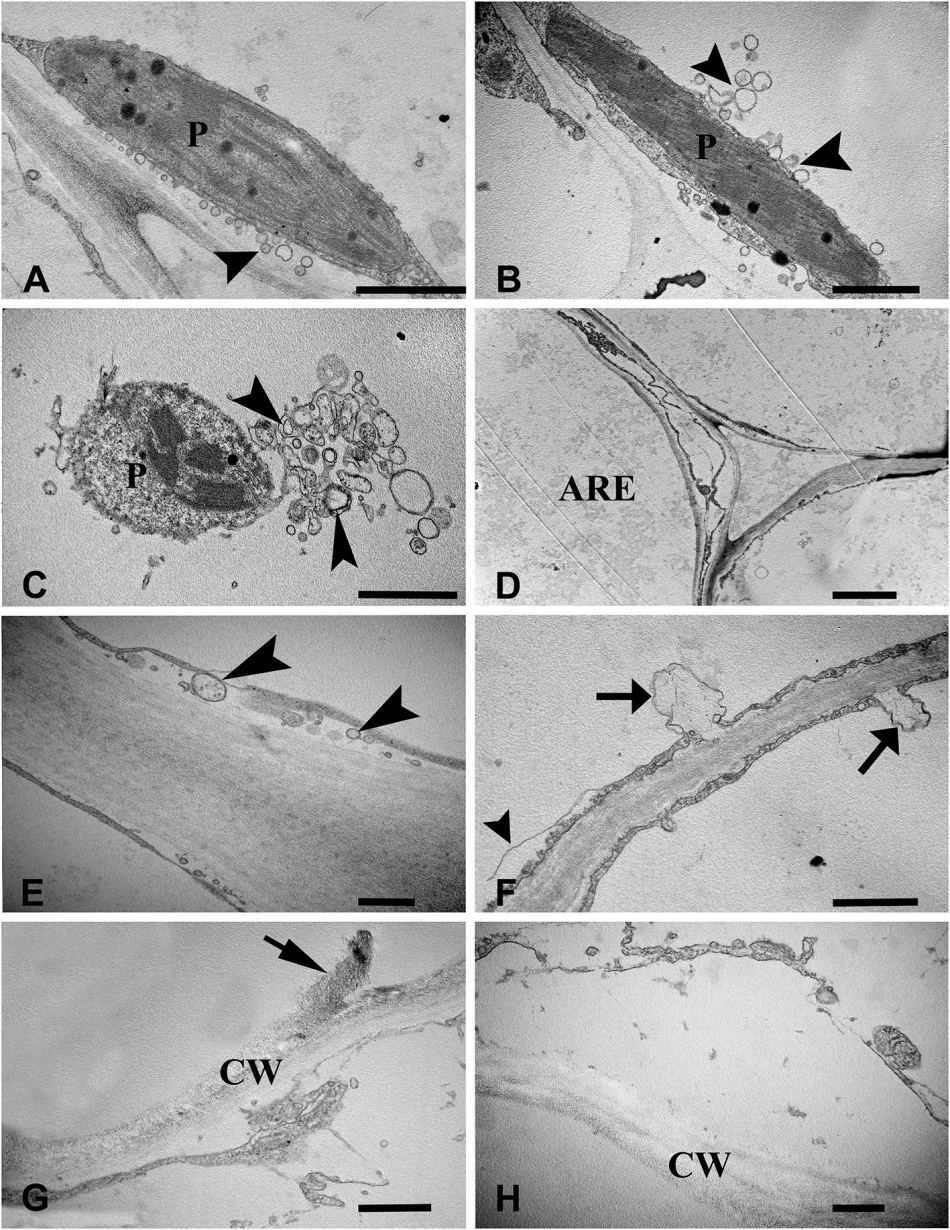
Ultrastructure of aerenchyma formation at late phase in *H. annuus* stems. **(A)** At the third day of WA treatment, many secretory vesicles (arrowhead) were observed around the plastid. **(B)** The plastids thylakoids became indistinct, and the number of secretory vesicles (arrowhead) decreased. **(C)** Plastid degradation. **(D)** The cell around the aerenchyma became flattened and the two sides of the collapsing cell wall was nearly aggregated together. **(E)** Vesicles containing granule materials were released into the interlayer between the plasma membrane and cell wall (arrowhead). **(F)** At the fourth day of WA treatment, plasma membrane invagination (arrow) and rupture (arrowhead) were observed. **(G)** Cell wall collapse (arrow). **(H)** The broken cell walls became thin and transparent. There are three different pictures was analyzed for each state. Bar: **(A–C,F)** = 1 μm, **(D)** = 2.5 μm, the others = 0.5 μm.

To examine the ultrastructural characteristics of aerenchyma formation in the stem, we used TEM to observe the cytological changes in the aerenchyma cavity at the different developmental stages. The cortical cells of control plants (without WA treatment) showed a normal ultrastructure with intact cell walls and cytoplasm (Figures 2A,B). When the plants were subjected to WA, the cortical cells at the early pre-cavity stage displayed visible changes in morphology, and plasmolysis and cell wall infolding were also observed (Figures 2C,D). After 1 day of WA, increased chromatin condensing was observed in the nuclei, and the nuclear envelope was heavily stained (Figure 2E). Nuclear invagination and nuclear envelope rupture were observed as hallmarks of nuclei degradation (Figure 2F). Subsequently (after 2 days of WA), ruptured tonoplasts were apparent, and a large number of vesicles, occasionally containing organelle material, were observed in the cytoplasm (Figure 2G). Characteristic membranous structures, such as multilamellar structures, were observed in the cells around the periphery of the developing aerenchyma, in which some vesicles, with or without contents, were enveloped (Figure 2H). In the expansion cavity stage (at the third day of WA), the plastid showed intact, but disturbed with lots of vesicles (Figure 3A) and indistinct thylakoids (Figures 3B,C). At the end of this stage, the cytoplasm was almost completely degraded, and the walls of the collapsing cells were nearly aggregated together (a diameter of 1–2 μm), leaving a large aerenchyma cavity in the cortex (Figure 3D). In addition, vesicles containing granule materials were released into the interlayer between the plasma membrane and cell wall (Figure 3E). At the fourth day of WA, Characteristics of plasma membrane invagination and rupture were obviously observed (Figure 3F). Cell wall collapse was the last cytological event of PCD in the process of aerenchyma formation, and the damaged cell walls appeared thin and transparent (Figures 3G,H).

### DNA Cleavage During Lysigenous Aerenchyma Formation

**Figure 4:**
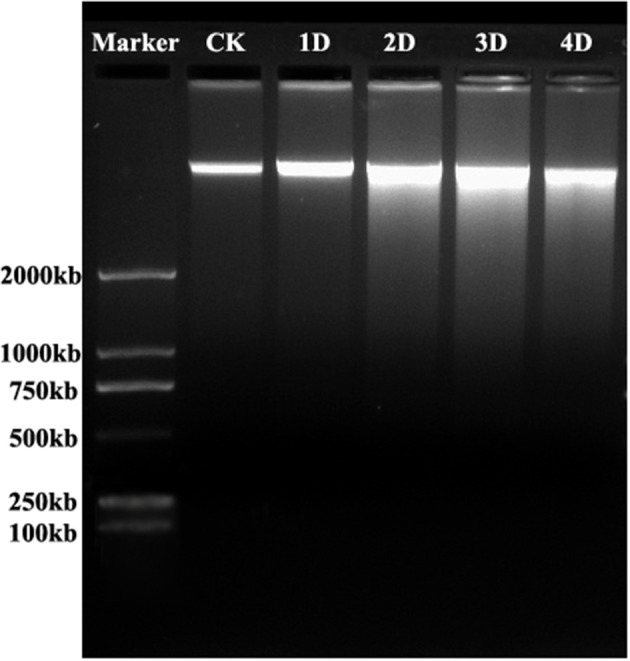
DNA cleavage during aerenchyma formation in *H. annuus* stems. Genomic DNA was isolated from control (lane 1) and waterlogged seedlings after 1D (Lane 2), 2D (Lane 3), 3D (Lane 4), and 4D (Lane 5), stained with ethidium bromide, and electrophoretically separated. Lanes 1 and 2. DNA ladder was not detected. Lanes 3 and 4: Clear DNA smearing present on the second and third day after WA treatment, it shows a number of cells undergo PCD in thses stages; Lane 5: Light DNA smearing present on the fifth day after WA treatment. Consistent results were obtained in three independent extracted DNA samples. Marker: 2000 bp.

**Figure 5:**
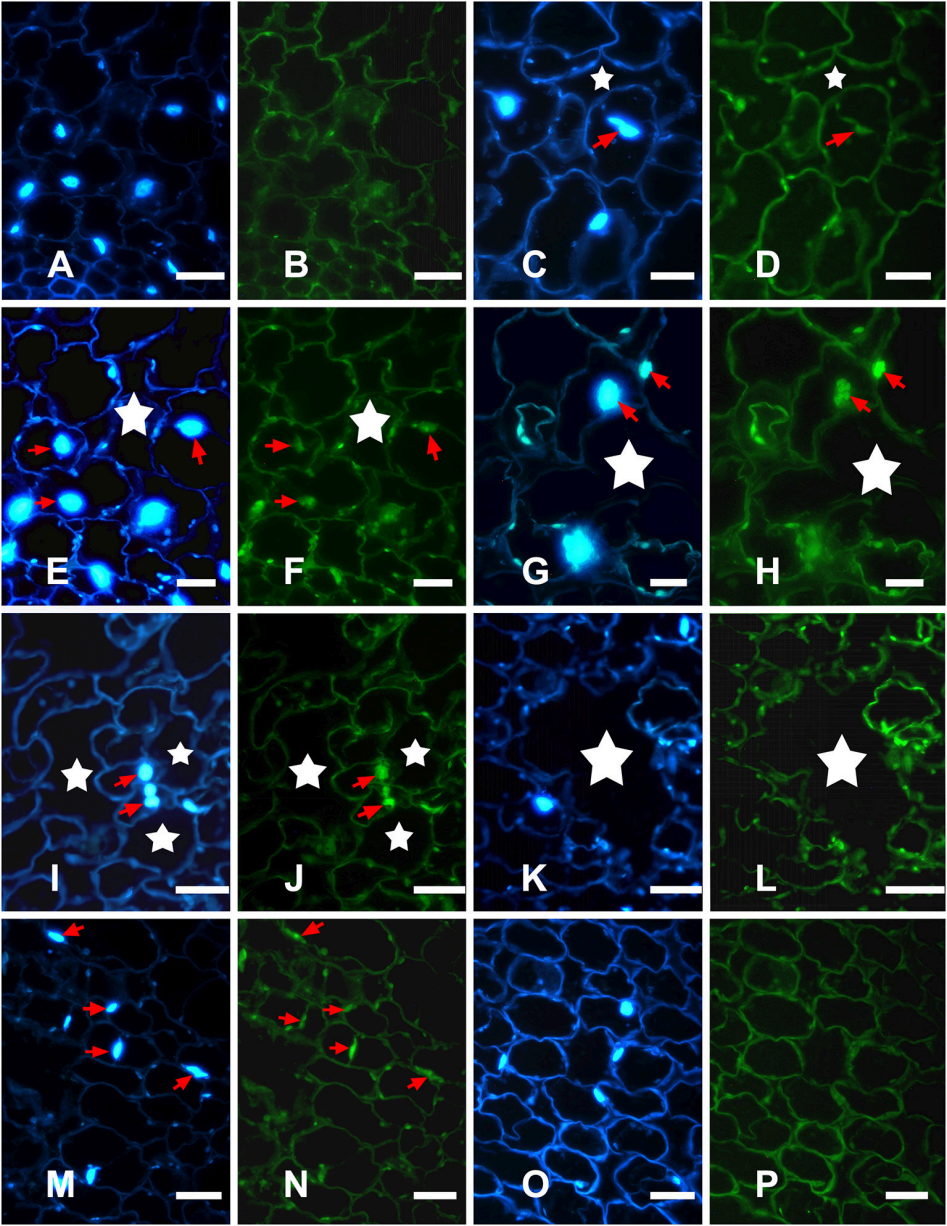
Detection of *in situ* DNA fragmentation in *H. annuus* stems using DAPI staining and TUNEL assays. **(A,C,E,G,I,K,M,O)** DAPI assays for nuclear changes; **(B,D,F,H,J,L,N,P)** TUNEL assays for nuclear DNA fragmentation. **(A,B)** Control plants showing no aerenchyma and DAPI-positive and TUNEL-negative staining. **(C,D)** In early phase of aerenchyma formation (star), TUNEL-positive nuclei were first detected in certain cells of the cortex. **(E,F)** In the formation phase of aerenchyma formation (star), TUNEL-positive nuclei were observed around the intercellular space; **(G–J)** In the expansion phase of aerenchyma formation (star), the remaining cells surrounding the lacunae showed a high frequency of TUNEL-positive nuclei. **(K,L)** In the mature phase of aerenchyma formation (star), no TUNEL-positive nuclei were observed in the stem tissues; **(M,N)** TUNEL-positive control; **(O,P)** TUNEL-negative control.

To examine nuclear DNA cleavage, a hallmark of PCD, during aerenchyma formation in *H. annuus*, genomic DNA was isolated from the stems, which was waterlogged from 1 to 4 days, as well as the control. The isolated DNA was analyzed by agarose gel electrophoresis (Figure 4). Beginning after 1 day of WA, a faint DNA smear was detected and after 2 days, DNA smearing was conspicuous. A more precise identification of PCD was achieved using TUNEL assays and DAPI staining. No labeling was observed in any part of the control plant stem (Figures 5A,B). However, TUNEL-positive nuclei were first detected in certain cells of the cortex at the early phase of aerenchyma formation (Figures 5C,D), revealing the onset of DNA cleavage, consistent with the detection of DNA gel electrophoresis. The labeling of the nuclei in the cells around the intercellular space was obvious at the formation phase of aerenchyma formation (Figures 5E,F), and progressively increased at the expansion phase of aerenchyma formation (Figures 5G–J). At the mature phase, aerenchyma was formed through the surrounding cells degeneration, and no TUNEL-positive nuclei were detected in the stem cross-sections (Figures 5K,L). For the positive control, almost all of the cells were TUNEL positive, which was treated with DNase I (Figures 5M,N). No positive labeling was observed in the negative controls (Figures 5O,P).

### The Effect of Waterlogging, Ethylene, and ROS in Aerenchyma Formation in *H. annuus* Stems

**Figure 6:**
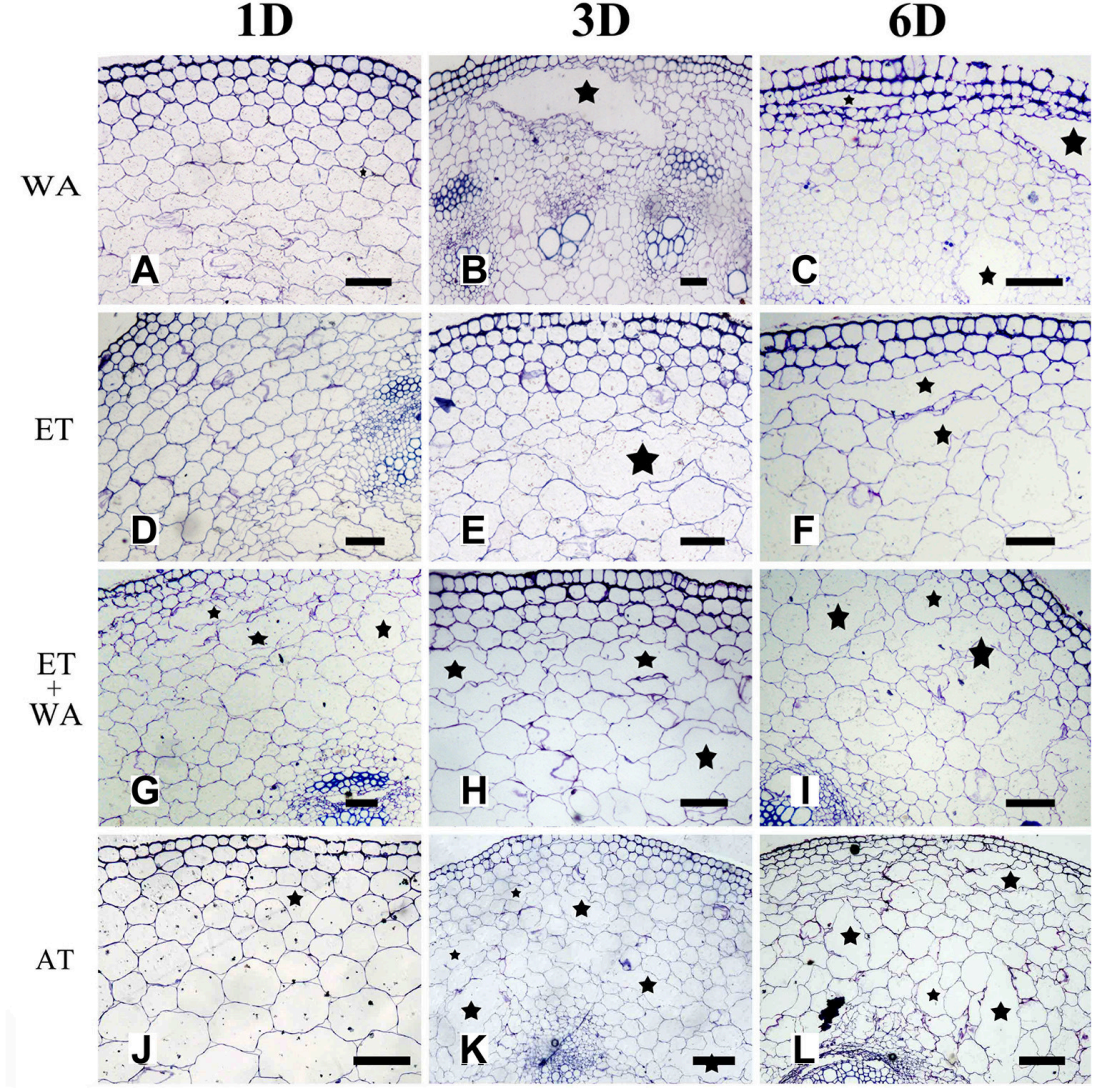
The effect of WA, ET, and AT on aerenchyma induction in *H. annuus* stem. **(A–C)** *H. annuus* seedlings were treated with WA; **(A)** Treat for 1 day; **(B)** Treat for 3 days; and **(C)** Treat for 6 days. **(D–F)** *H. annuus* seedlings were treated with ET; **(D)** Treat for 1 day; **(E)** Treat for 3 days; and **(F)** Treat for 6 days. **(G–I)** *H. annuus* seedlings were treated with ET + WA; **(G)** Treat for 1 day; **(H)** Treat for 3 days; and **(I)** Treat for 6 days. **(J–L)** *H. annuus* seedlings were treated with AT; **(J)** Treat for 1 day; **(K)** Treat for 3 days; and **(L)** Treat for 6 days. The stars in figure represent for the cavities of aerenchyma formation. Bars = 50 μm.

**Figure 7:**
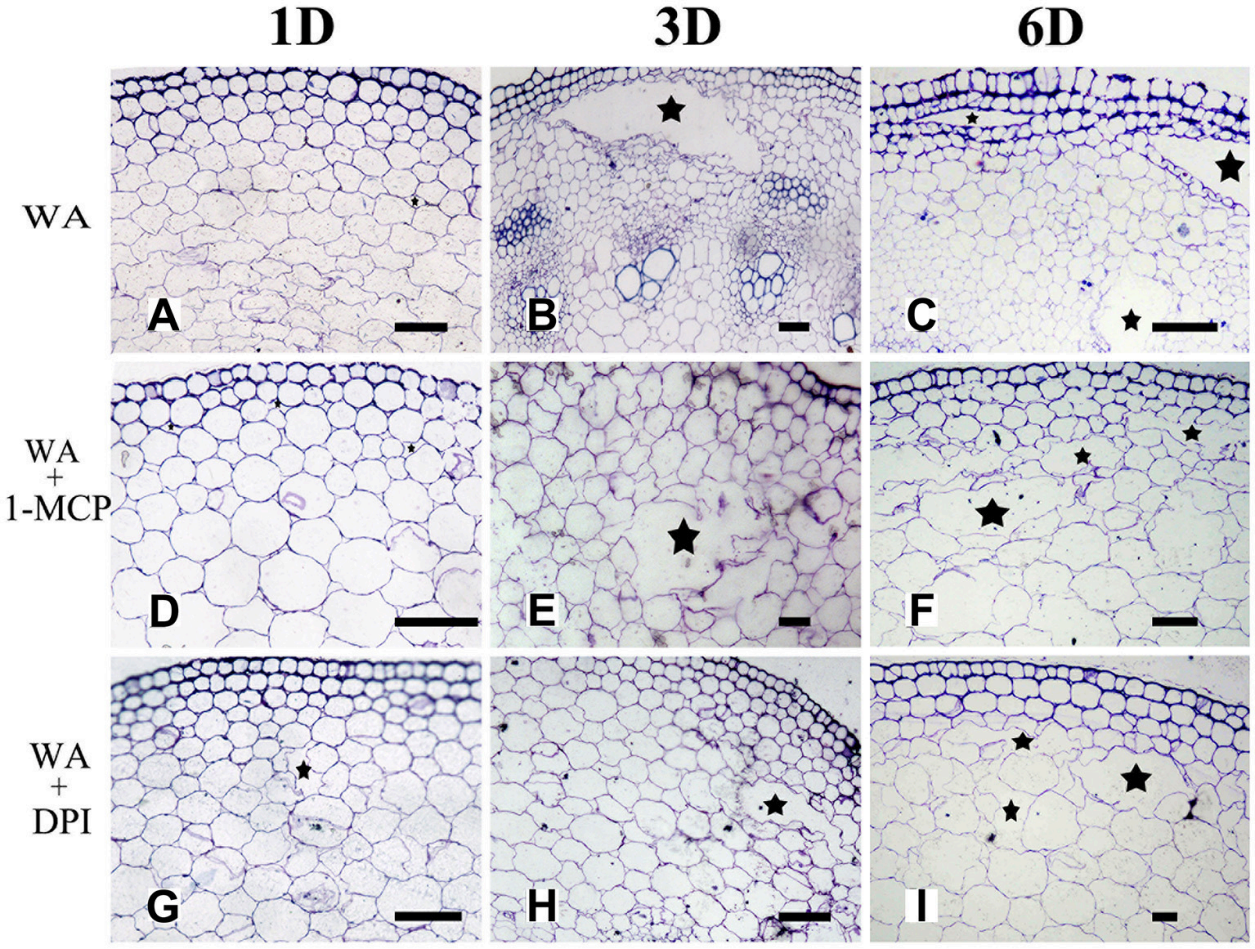
The effect of WA on aerenchyma induction or inhibition in *H. annuus* stem. **(A–C)** *H. annuus* seedlings treated with WA; **(A)** Treat for 1 day **(B)** Treat for 3 days; and **(C)** Treat for 6 days; **(D–F)** *H. annuus* seedlings treated with WA + 1-MCP; **(D)** Treat for 1 day; **(E)** Treat for 3 days; and **(F)** Treat for 6 days; **(G–I)** *H. annuus* seedlings treated with WA+DPI; **(G)** Treat for 1 day; **(H)** Treat for 3 days; and **(I)** Treat for 6 days. The stars in figure represent for the cavities of aerenchyma formation. Bars = 50 μm.

**Figure 8:**
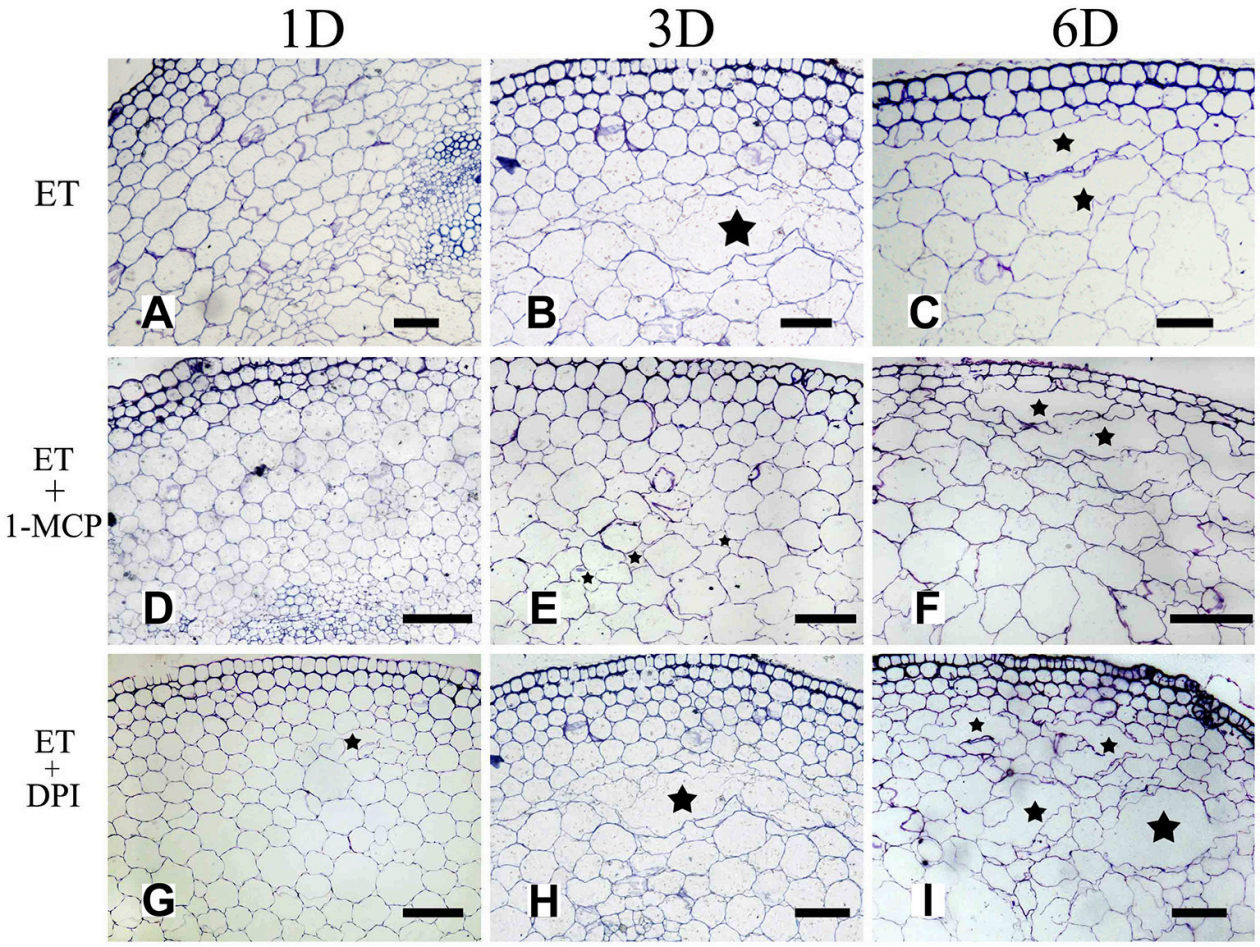
The effect of ET on aerenchyma induction or inhibition in *H. annuus* stem. **(A–C)** *H. annuus* seedlings treated with ET; **(A)** Treat for 1 day **(B)** Treat for 3 days; and **(C)** Treat for 6 days; **(D–F)** *H. annuus* seedlings treated with ET + 1-MCP; **(D)** Treat for 1 day; **(E)** Treat for 3 days; and **(F)** Treat for 6 days; **(G–I)** *H. annuus* seedlings treated with ET+DPI; **(G)** Treat for 1 day; **(H)** Treat for 3 days; and **(I)** Treat for 6 days. The stars in figure represent for the cavities of aerenchyma formation. Bars = 50 μm.

**Figure 9:**
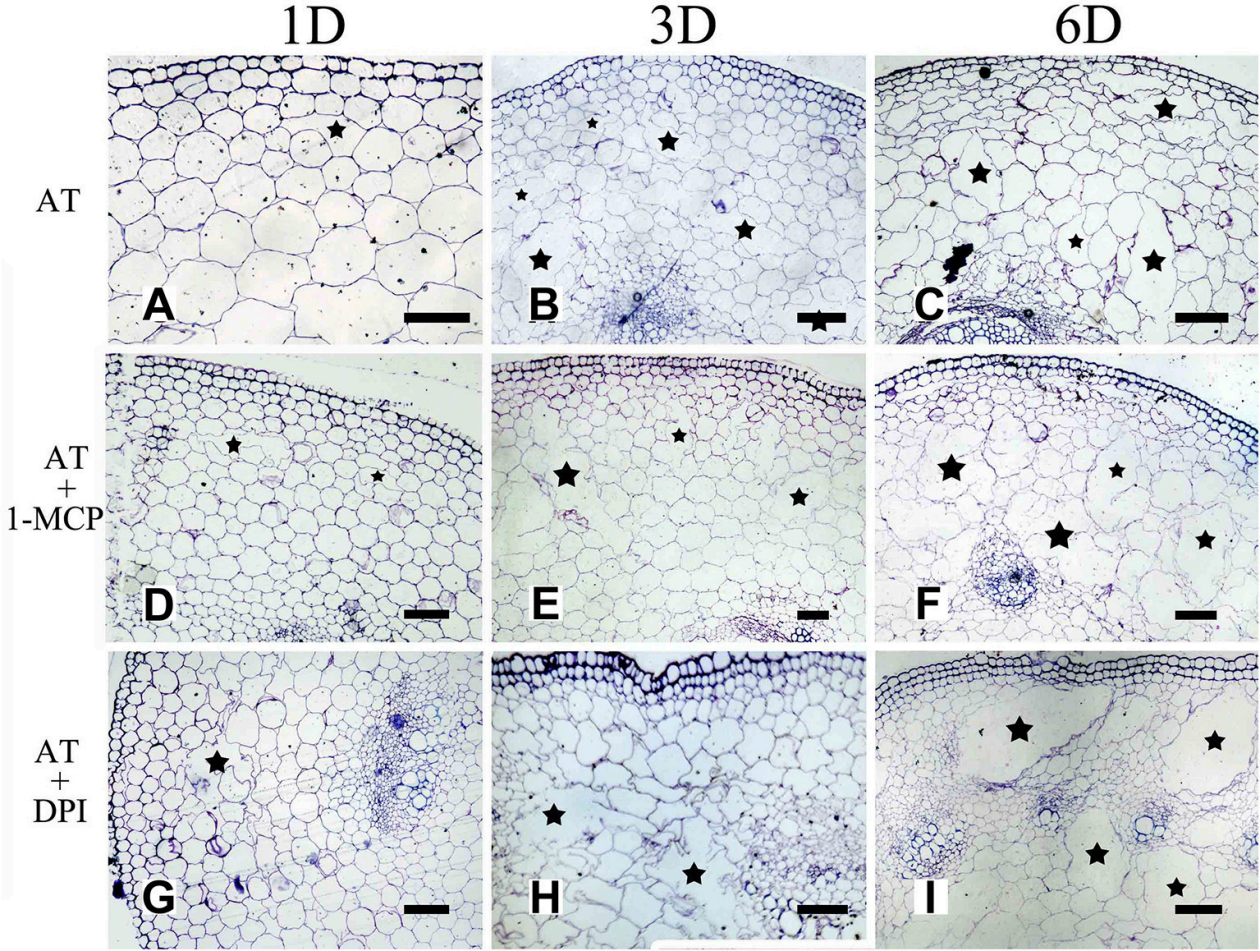
The effect of ROS on aerenchyma induction or inhibition in *H. annuus* stem. **(A–C)** *H. annuus* seedlings treated with AT; **(A)** Treat for 1 day, **(B)** Treat for 3 days; and **(C)** Treat for 6 days; **(D–F)** *H. annuus* seedlings treated with AT + 1-MCP; **(D)** Treat for 1 day; **(E)** Treat for 3 days; and **(F)** Treat for 6 days; **(G–I)** *H. annuus* seedlings treated with AT+DPI; **(G)** Treat for 1 day; **(H)** Treat for 3 days; and **(I)** Treat for 6 days. The stars in figure represent for the cavities of aerenchyma formation. Bars = 50 μm.

**Figure 10:**
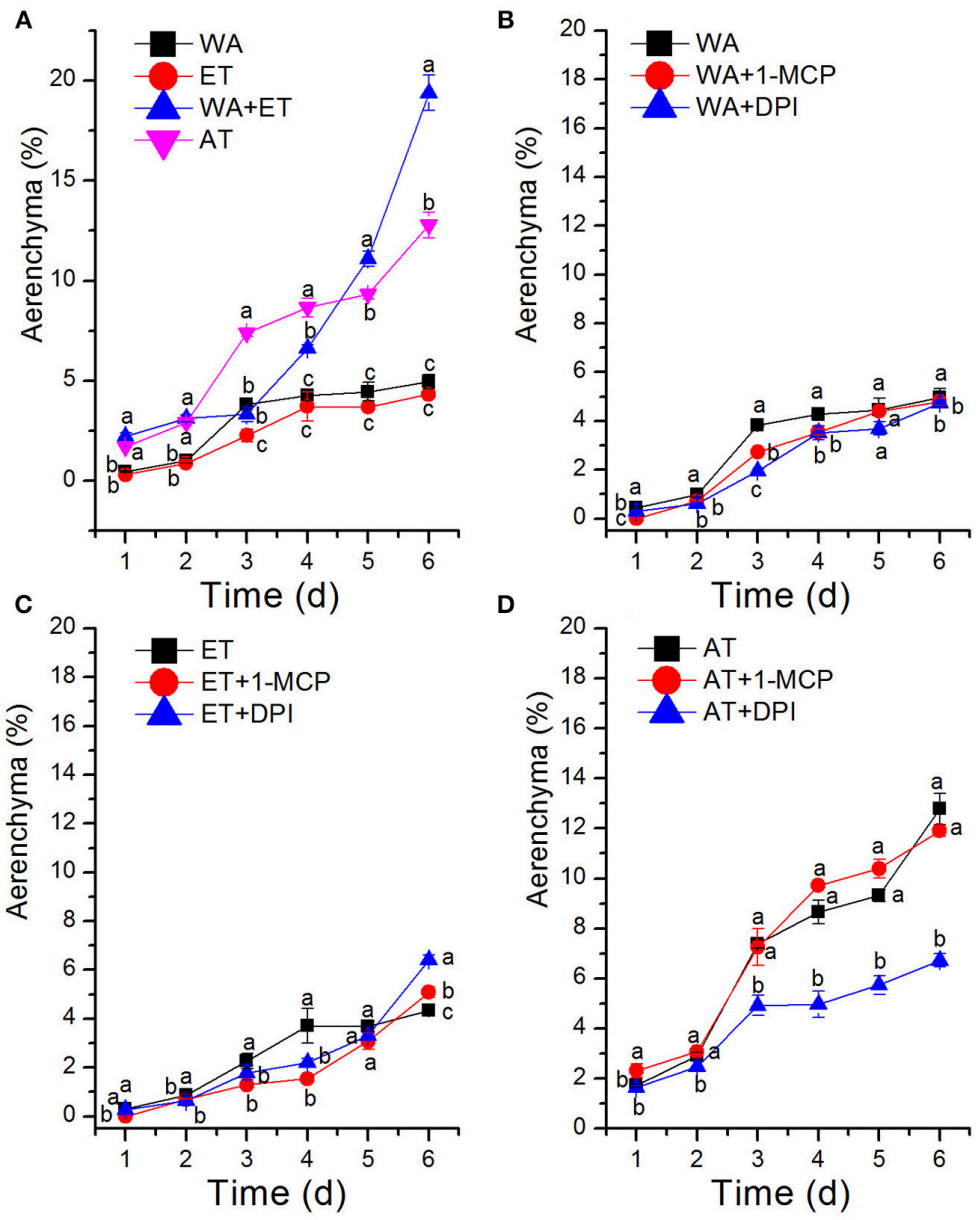
Statistical analysis of aerenchyma area after treatment with ethylene, reactive oxygen species and their inhibitors. **(A)** Aerenchyma formation in *H. annuus* seedlings was induced through WA, ET, AT, and combined treatment with ET & WA. **(B)** Aerenchyma formation was induced through WA, with or without pre-treatment with 1-MCP or DPI. **(C)** Aerenchyma formation was induced through ET, with or without pre-treatment with 1-MCP or DPI. **(D)** Aerenchyma formation was induced through AT, with or without pre-treatment with 1-MCP or DPI. Data shown are average and SD for 3 biological replicates. The different lower case letters stand for different significance among the treatments.

Previous studies have shown that oxygen deficiency triggers ethylene production in plant, and result in aerenchyma formation in waterlogged tissue of plant (Kawase, 1981; He et al., 1992; Dauphinee et al., 2012). To further understand the role and relationship of different signals in the regulation of aerenchyma formation, we analyzed the short-term time sequence of aerenchyma formation in the stems of *H. annuus* seedlings treated with WA, ET or ROS (Figures 6–**9**). In our study, the treatment of ET to induce aerenchyma formation in sunflower stem was used by spraying 150 μM ethephon. The treatment of ROS to induce aerenchyma formation was used by spraying 50 mM AT, as the catalase inhibitor to promote ROS accumulation in the cells. Treatment with WA, ET, or ROS or the combination of ET & WA induced a gradual increase in the amount of aerenchyma formation in the stem, which was expressed as the percentage of aerenchyma area in the cross-section of stem (Figures 6, **10A**, Table 1). This was in contrast to the seedlings that were untreated, which barely formed aerenchyma during the course of normal growth and development. The highest amount of aerenchyma formation was observed after 6 days of combined treatment of ET & WA. AT induced the higher amount of aerenchyma formation than WA, whereas ET induced the lowest amount of aerenchyma formation. Oneway ANOVA revealed significant differences between these treatments (*P* < 0.05). Treatment with AT and the combination of ET & WA induced aerenchyma formation on the first day, but aerenchyma formation at the same stage was minimal after treatment with ET or WA alone (Figure **10A**).

To determine whether WA-induced aerenchyma formation is independent of ET or ROS signals, *H. annuus* seedlings were pre-treated with 1-MCP (ethylene perception inhibitor) or DPI (NADPH oxidase inhibitor, to decrease endogenous ROS levels), followed by treatment with WA, and seedlings without pre-treatment were used as a control (Table 2). The results showed that at the same stage, 1-MCP pre-treatment or DPI pre-treatment decreased the amount of aerenchyma formation compared with waterlogged seedlings (CK) (Figures 7, **10B**). By the third day, the amount of aerenchyma formation in CK reach to 3.82%, while the amount of aerenchyma formation in 1-MCP pre-treatment or DPI pre-treatment seedlings decreased to 2.72 and 1.95% respectively. A oneway ANOVA of the amount of aerenchyma formation shows that during the whole period of treatment except for the fifth day, there are significant differences between the CK and 1-MCP pre-treatment or DPI pre-treatment seedlings (*p* < 0.05) (Figure **10B**).

To further determine the interrelationship between ET and ROS in regulating aerenchyma formation, pre-treated with inhibitors (1-MCP or DPI), one group of the seedlings was treated with ET, and seedlings without pre-treatment were used as a control (Table 3). The seedlings pre-treated with inhibitors exhibited a lower amount of aerenchyma formation than the control (Figures 8, **10C**), especially in the treatment with 1-MCP + ET, aerenchyma formation was minimal observed in the first day (Figure **10C**). By the fourth day, the amount of aerenchyma formation in control seedlings increased to 3.72%, while the amount of aerenchyma formation in 1-MCP pre-treatment or DPI pre-treatment seedlings was 1.54 and 2.22% respectively (Figure **10C**). A oneway ANOVA revealed significant differences in the amount of aerenchyma formation (in all but the fifth day), between the pre-treated and control seedlings (*P* < 0.05). No significant differences were observed between 1-MCP pre-treatments and DPI pre-treatments (*P* > 0.05). Another group of the seedlings were subsequently treated with AT, and seedlings without pre-treatment served as a control (Table 4). Seedlings pre-treated with DPI inhibitors showed obviously lower amount of aerenchyma than the control seedlings (Figures 9, 10D). By the sixth day, the amount of aerenchyma formation was 12.77% in control seedlings, compared to 6.72% in DPI pre-treated seedlings. A oneway ANOVA revealed significant differences between the DPI pre-treatment and control (*P* < 0.05). However, aerenchyma formation was comparable between the control group and seedlings pre-treated with the inhibitor 1-MCP. A oneway ANOVA revealed no significant differences observed between the 1-MCP pre-treatment and control (*P* > 0.05) (Figures 7G–L, 10D).

## Disscussion

### PCD Is Involved in Waterlogging-Induced Aerenchyma Formation in *H. annuus* Stems

Kawase (1974, 1981) and Kawase and Whitmoyer (1980) described aerenchyma development in the cortex of waterlogged or ethylene-induced *H. annuus* stems and roots. Yet, the cellular and molecular mechanisms associated with PCD during aerenchyma formation in *H. annuus* were poorly understood. In the present study, we conducted a detailed investigation of the cytological events of PCD during aerenchyma formation using light and transmission electron microscopy, TUNEL assays and DNA gel electrophoresis.

Plasmolysis was initially observed in cells undergoing PCD, consistent with the plasma membrane retraction from the cell wall previously observed during aerenchyma formation in waterlogged maize roots. Subsequently, cell wall infolding was observed, reflecting protoplast degradation, and the volume of the cell shrinkage. Apparently concurrent with the change of nuclear morphology, some notable features of nuclear degradation, including chromatin condensation (Figure 2E), nuclear invagination and nuclear envelope rupture (Figure 2F), were observed in cells undergoing PCD. Cleavage of genomic DNA into smaller fragments was a remarkable cytological feature of PCD (Gray, 2004; Reape and McCabe, 2008). However, a DNA smearing was clearly identified in sunflower stems, which waterlogged at different days (Figure 4). Most of the time, the DNA electrophoresis did not show “ladders” during the PCD process in plants, rather it showed a DNA smear (Ni et al., 2015). These characteristics were also reported during aerenchyma formation in *Typha angustifolia* leaves (Ni et al., 2014) and are consistent with vascular cavity formation in *Pisum sativum* roots (Sarkar and Gladish, 2012), the wilting of *Antirrhinum majus* flower petals (Yamada et al., 2006), and so on. The process of PCD was also verified using a TUNEL assay, based on the intense labeling of the nuclei in the cells of these tissues, that cells undergo PCD at the early phase of aerenchyma formation (Figure 5). Extensive TUNEL-positive nuclei in the waterlogged seedlings on the third day, reflected occurrence of PCD in late developed aerenchyma.

Following this process, some remarkable features of PCD include tonoplast rupture and vesicle formation (Figure 2G). The observed vesicles were double-membraned vesicular structures that occasionally contained organelle materials (Figure 2H). The same phenomenon has been observed in either developmental or induced PCD in other plants (Gunawardena et al., 2001a; Bozhkov et al., 2005; Liu et al., 2005, 2010; Domínguez et al., 2012; Wertman et al., 2012).

Concerning organelle degradation, plant biologists has been primarily concerned with mitochondria, the endoplasmic reticulum and the Golgi apparatus (Gunawardena et al., 2001a; Jiang et al., 2011; Domínguez et al., 2012; Eichmann and Schäfer, 2012; Mulisch et al., 2013; Jiao et al., 2014). The results presented herein provide additional evidence of plastid degradation during PCD, as secretory vesicles and indistinct thylakoids were observed (Figures 3A,B). The cell wall degradation is the last event in PCD (Figures 3G,H). Numerous vesicles were observed to be transported outside of the plasma membrane, close to the cell wall, which might play a role in cell wall degradation, as observed in *T. angustifolia leaves* (Ni et al., 2014).

### Ethylene Signaling Is a Crucial Factor Involved in Aerenchyma Formation

Flooding affects gas exchange between the shoot and the waterlogged roots, and results in ethylene accumulated rapidly in submerged tissue of plants (Steffens and Sauter, 2014). The role of ethylene accumulated in submerged tissue can mediates many adaptative characteristics to submergence, such as adventitious root and aerenchyma formation to avoid oxygen deficiency under flooded conditions (Hu et al., 2016). The present study indicated that WA and ET signaling are involved in aerenchyma formation, which was consistent with the results of Kawase (1981). We also identified that the aerenchyma initially formed in the middle of the cortex in the stem (Figures S1A,C), subsequently formed in the exodermis and occasionally connects to the stoma of the stem (Figures S1B,F) to facilitate gas exchange, and ultimately formed in the pith and vascular bundles (Figures S1D,E). This suggests that hypoxia induced aerenchyma formation in different tissues is possibly a beneficial stress adaptation of sunflower seedlings. It has been reported that aerenchyma formation in the vascular bundles and pith has pay out heavy cost to meet the respiratory demands of developing cells and survive from extremely hypoxic stress by sacrificing such important cells (Lu et al., 1991). Furthermore, aerenchyma formation not only improves gas exchange, but also reduces the number of cells requiring oxygen for respiration (Zhu et al., 2010; Postma and Lynch, 2011). Similar results of aerenchyma formation in recombinant inbred maize lines with improved drought tolerance might also result from cavity formation reduces metabolic cost by reducing cell numbers of the roots (Zhu et al., 2010). Similarly, aerenchyma formation may be an adaptation to survive from hypoxic stress by sacrificing important cells in waterlogged sunflowers.

Furthermore, the combined treatment of ET & WA induced a higher amount of aerenchyma formation (Figure 10A). In addition, the amount of aerenchyma formation was reduced under waterlogging or ET treatment condition when the ET signal pathway was blocked by ethylene perception inhibitor 1-MCP (Figures 10B,C). This result is consistent with a study in ACC pre-treatment wheat seedlings enhanced aerenchyma formation to adapt stagnant conditions (Yamauchi et al., 2014). Promotion of induction of aerenchyma formation by ethylene, either endogenously produced, or exogenously applied, has also been reported in the roots of some plants, such as in maize (Gunawardena et al., 2001a), rice (Steffens et al., 2011), *Dendranthema* spp. (Yin et al., 2013), and wheat (Yamauchi et al., 2014). At the same time, aerenchyma has been shown to be prevented by inhibitors of ethylene action (Drew et al., 1981; Yin et al., 2013). Steffens et al. (2011, 2012) reported that aerenchyma formation in rice is enhanced by waterlogging and by ET. In related research of ACS gene expression and the rate-limiting step in the ethylene biosynthesis gene, it was reported that the ACS gene expressed itself both in the root and shoot of arabidopsis when the roots were immersed in media with 3% oxygen (Peng et al., 2005). These achievements further support that ethylene is involved in the signaling pathway of aerenchyma formation in sunflower stem.

### ROS Is Essential for Ethylene-Induced Aerenchyma Formation

PCD in plants is often mediated by signaling molecules, and it has been report that the ROS, ${\text{O}}_{2}\cdot^{-}$ and H_2_O_2_ are central regulators of PCD (Moeder et al., 2002; Overmyer et al., 2003; Bouchez et al., 2007). However, the interrelationship between ethylene and ROS during aerenchyma formation in *H. annuus* is not completely unraveled yet. In the current study, AT-induced seedlings showed a higher amount of aerenchyma formation compared with WA- and ET-induced seedlings. ET-induced aerenchyma formation was partially suppressed using the ethylene inhibitor, 1-MCP, and the NADPH oxidase inhibitor, DPI (Figures 10B,C). AT-induced aerenchyma formation was also significantly inhibited through DPI pre-treatment, but this process was only slightly inhibited through 1-MCP pre-treatment (Figure 10D). These results clearly indicated a causal and interdependent relationship between WA, ET and ROS signaling in aerenchyma formation. Therefore, it is apparent that ethylene- mediated ROS signaling plays a role in aerenchyma formation in *H. annuus* stems. This conclusion is supported by a study in rice showing that ET promotes ${\text{O}}_{2}\cdot^{-}$ formation in pre-aerenchymal cells, and H_2_O_2_ production and MT2b (the H_2_O_2_ scavenger) genetic downregulation promotes aerenchyma formation (Steffens et al., 2011). Lysigenous aerenchyma formation in maize roots (Rajhi et al., 2011) and wheat (Yamauchi et al., 2014) are also regulated through ROS signaling. Yamauchi et al. (2017) identified ethylene-dependent aerenchyma formation in rice roots, and further reported that RBOH gene expression and ROS accumulation were essential for ethylene-induced aerenchyma formation under oxygen-deficient environment. Similarly, the induction of lysigenous aerenchyma formation in wheat roots is regulated through ET and the ROS signaling pathway (Yamauchi et al., 2014). In addition, the exogenous application of H_2_O_2_ promoted the death of epidermal cells above nodal adventitious root primordia, even in the presence of 1-MCP, indicating that H_2_O_2_ acts downstream of ethylene (Steffens and Sauter, 2009). Therefore, ROS signal is crucial for ethylene-dependent aerenchyma formation.

In summary, we conclude that PCD is involved in aerenchyma formation in *H. annuus* stems under waterlogging. Ethylene and ROS play a role in inducing the formation of lysigenous aerenchyma in the stems, and ROS is essential for ethylene-induced aerenchyma formation in *H. annuus* stems. These results will no doubt provide further insight into the role and interrelationship of WA/hypoxia, ET and ROS in PCD-regulated signal networks. However, little is known about the downstream signaling and molecular regulatory mechanisms of ROS during plant PCD; indeed, transcriptome analyses and related genes expression should be further studied in the future.

## Author Contributions

X-LN and W-ZL designed the study. C-XL developed the scaling theory. XL-N and M-YG performed the waterlogging experiment. L-LT and QZ performed the ethylene and ROS experiment. X-LN wrote the first draft of the manuscript. All authors contributed to improve the manuscript.

### Conflict of Interest Statement

The authors declare that the research was conducted in the absence of any commercial or financial relationships that could be construed as a potential conflict of interest.

## Abbreviations

1-MCP: 1-Methylcyclopropene
AT: 3-amino-1, 2, 4-triazole
C: cavity
CW: cell wall
DPI: diphenyleneiodonium
ET: ethephon
G: golgiosome
M: mitochondria
N: nucleus
P: plastid
PCD: programmed cell death
PT: palisade tissue
ROS: reactive oxygen species
ST: spongy tissue
V: vacuole
VB: vascular bundle
Ve: vesicle
WA: waterlogging.
