# Supplementary material for: Programmed Cell Death and Aerenchyma Formation in Water-Logged Sunflower Stems and Its Promotion by Ethylene and ROS
Source: Front Plant Sci. 2019 Jan 9;9:1928. doi: 10.3389/fpls.2018.01928 (PMC6333753; doi:10.3389/fpls.2018.01928)
Supplement: Supplementary file 1 [file Data_Sheet_1.PDF]

Supplementary Figure 1

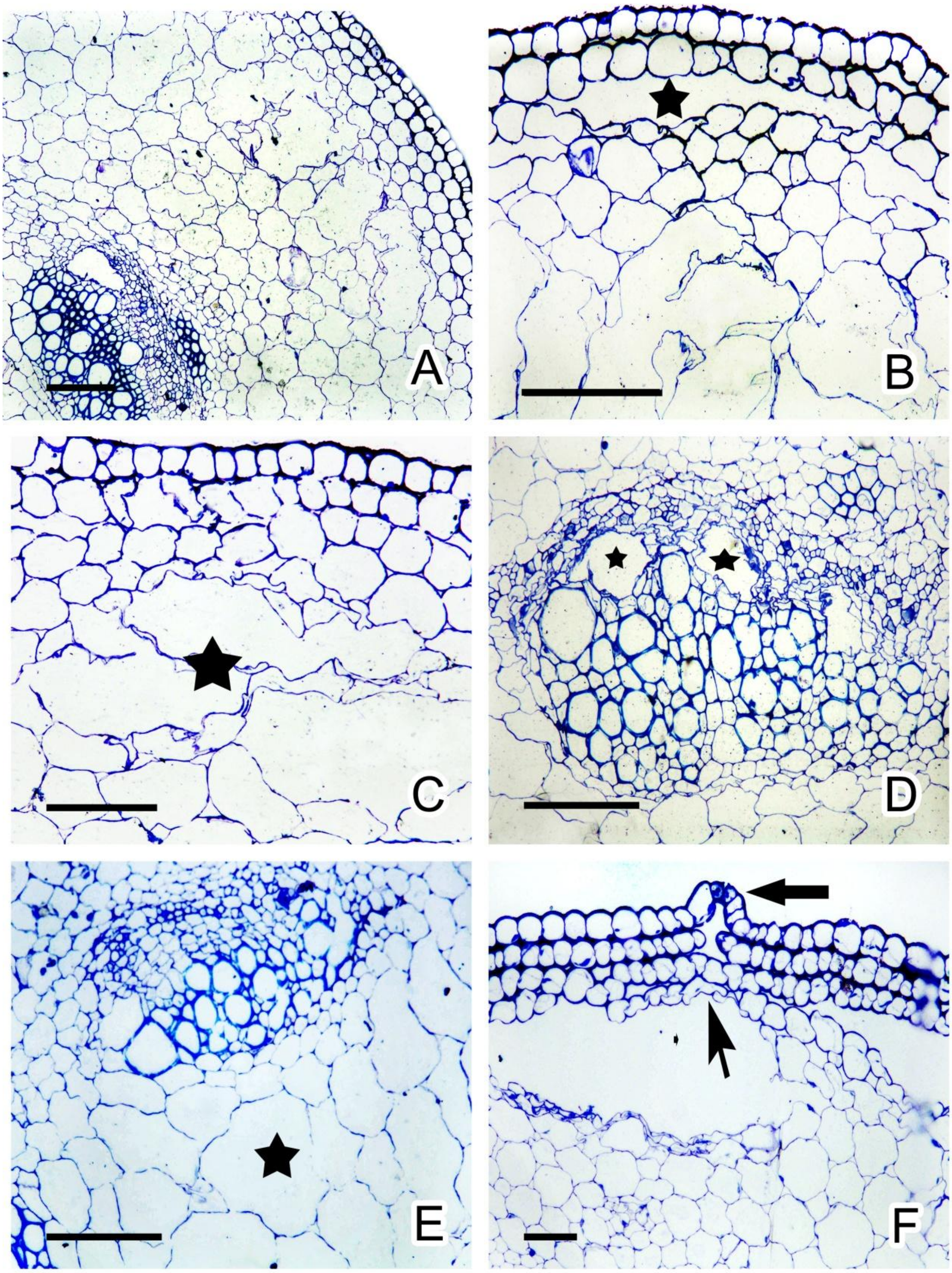

Figure S1. Distribution of waterlogging-inducted aerenchyma formation *H. annuus* stems.

A: Cross section an *H. annuus* stem, showing the distribution of aerenchyma; B: Distribution of aerenchyma in the exodermis; C: Distribution of aerenchyma in the cortex; D: Distribution of aerenchyma in the vascular tissue; E: Distribution of aerenchyma in the pith; and F: Relationship between aerenchyma formation and stoma distribution. Bars=50 μm.
